# Total percutaneous 4-vessel endovascular aortic arch repair with a triple inner-branch device and a fenestration

**DOI:** 10.1016/j.xjtc.2024.09.020

**Published:** 2024-10-02

**Authors:** Clément Willot, Jessica Forcillo, Jean-François Blair, Marie-Jo Plamondon, Stéphane Elkouri, Laura M. Drudi, Stephan Haulon, Philippe Charbonneau

**Affiliations:** a Aortic Center, Centre Hospitalier de l’Université de Montréal, Montreal, Québec, Canada; b Division of Vascular Surgery, Centre Hospitalier de l’Université de Montréal, Montreal, Québec, Canada; c Aortic Center, Hôpital Marie Lannelongue, Groupe Hospitalier Paris Saint-Joseph, Université Paris-Saclay, Paris, France

**Figure:**
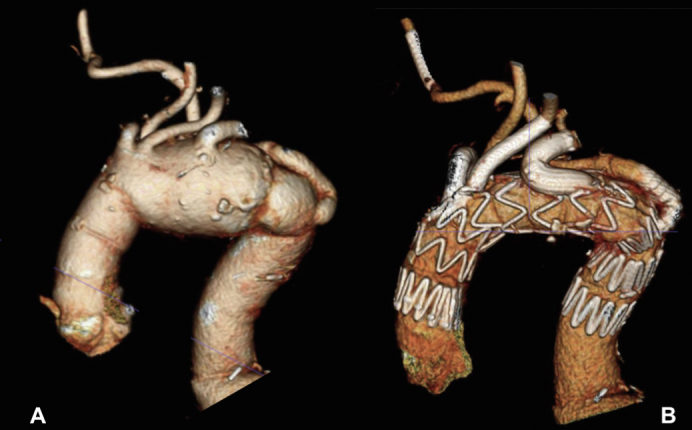
A, Aortic arch aneurism with aberrant subclavian artery. B, Four-vessel aortic arch repair.

**Central Message**

We report the first total percutaneous 4-vessel endovascular aortic arch repair with a triple inner-branch device and a laser fenestration for a complex arch aneurysm with an aberrant right subclavian artery.

Total endovascular aortic arch repair performed in expert centers has been proven to be safe and effective in patients with multiple comorbidities or those with contraindications to open surgery.^1^ When anatomic criteria are met, double and triple inner-branch endografts are the preferred designs. Totally percutaneous strategies have been associated with a reduction in access-related complications, thereby reducing subsequent reinterventions and stroke.^2^ We describe a total percutaneous 4-vessel endovascular aortic arch repair using a triple inner-branch custom-made device combined with an in situ laser fenestration for an arch aneurysm with an aberrant right subclavian artery (ARSA). Consent was obtained from the patient. Institutional review board approval was not required.

## Case Report

**Figure 1:**
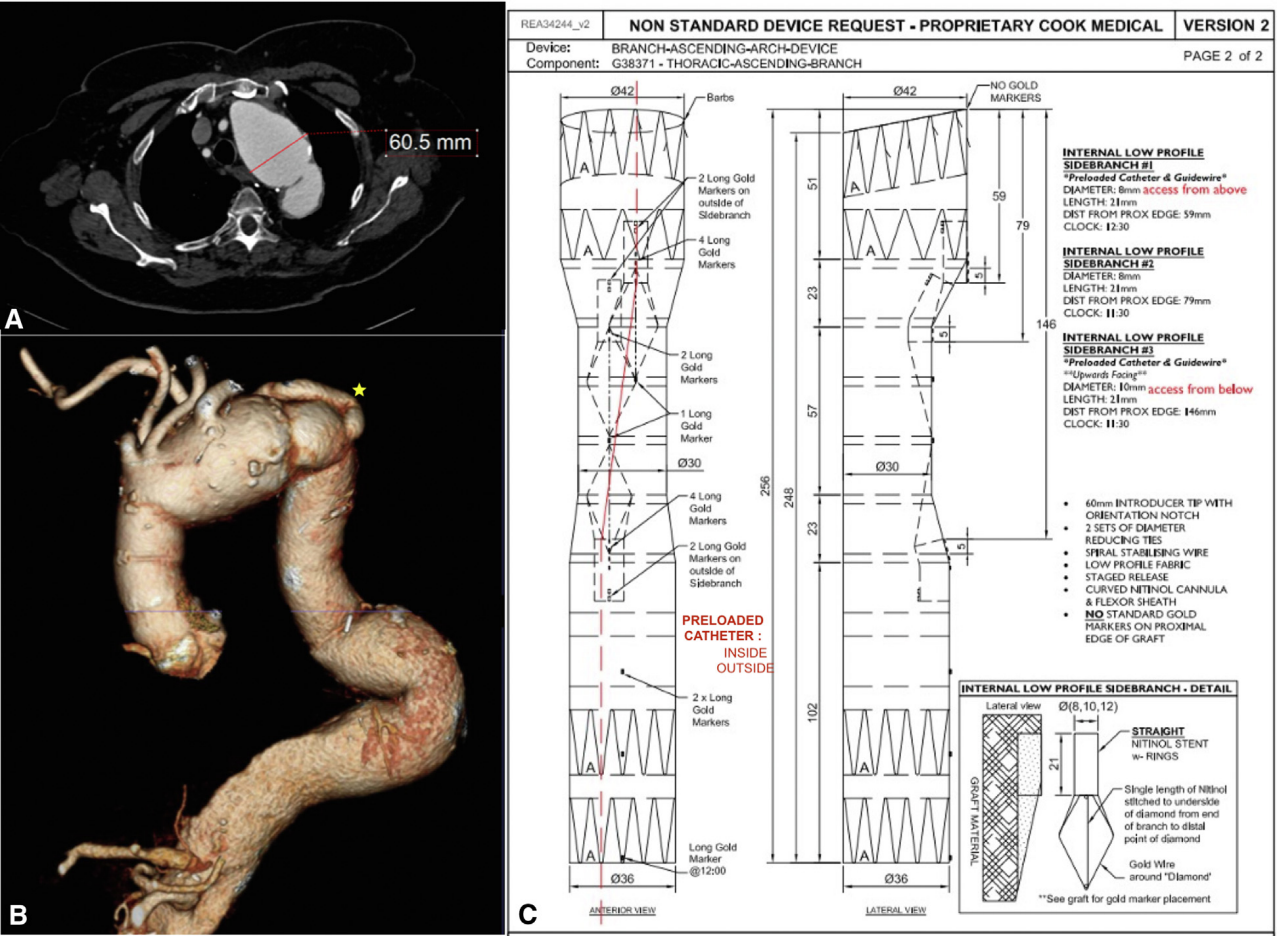
A, Three-dimensional computed tomography angiography demonstrated a 61-mm aortic arch aneurysm. B, Retroesophageal aberrant right subclavian artery (*yellow star*) was reimplanted on the descending thoracic aortic graft, with a posterior and kinked takeoff. C, Graft plan.

A 76-year-old woman was referred for an asymptomatic degenerative arch aneurysm. Her ascending and proximal descending thoracic aortas were surgically replaced 10 years earlier. Other relevant comorbidities include hypertension, asthma, and obesity (body mass index = 40). A computed tomographic angiography demonstrated a 61-mm aortic arch aneurysm. The ascending aortic graft was 37 mm in diameter and 53 mm in length. The right common carotid (RCCA), left common carotid (LCCA), and left subclavian artery (LSA) diameters were 8, 7.5, and 12 mm, respectively. A 12-mm diameter retroesophageal ARSA was reimplanted on the descending thoracic aortic graft, with a posterior and kinked takeoff (Figure 1, *A* and *B*). The proximal descending thoracic graft diameter was 34 mm. The aortic arch endograft used was a Cook custom-made device, which had 2 proximal anterograde and 1 retrograde inner branch for the RCCA, LCCA, and LSA, respectively. A preloaded access catheter was loaded in a retrograde fashion into the LSA branch and reentered the graft from the RCCA branch (Figure 1, *C*). To preserve the ARSA, an in situ laser fenestration was planned.

The procedure was performed under general anesthesia in a hybrid operating room (Video 1). Puncture was performed on the right common femoral artery (CFA) and 2 Prostyle devices (Abbott) were deployed. A left CFA and right axillary artery percutaneous accesses were obtained to introduce a 5 Fr and 6.5 Fr sheath, respectively. The branched-endograft was advanced over a Lunderquist wire across the aortic valve into the left ventricle from a 26 Fr × 65 cm Dryseal sheath (W.L. Gore & Associates). It was deployed above the sinotubular junction while we had temporary rapid pacing. A second Lunderquist wire was inserted into the preloaded catheter. The delivery system and the first Lunderquist wire were subsequently replaced with a 22 Fr × 65 cm sheath. We then performed a through-and-through access with a 0.014 wire from the preloaded catheter access, which was snared via the same right femoral sheath. A 6 Fr Cook shuttle sheath was advanced retrogradely via the RCCA branch and married into the dilator of an 8 Fr × 110 cm sheath. Using a pull-and-push maneuver, the 8 Fr sheath was advanced and positioned inside the RCCA branch. After the RCCA was catheterized, an Amplatz wire was inserted and 2 consecutive 8 × 57 mm BeGraft+ (Bentley InnoMed) stents were deployed. After RCCA control angiography, the through-and-through wire was retracted into the arch aneurysm and used to catheterize the second branch retrogradely. Then, the same steps for snaring were repeated for LCCA, and 28 × 57 mm BeGraft+ stents were deployed to bridge the branch to the artery. Finally, a 13 mm × 10 cm Viabahn (W.L. Gore & Associates) stent was used to bridge the third branch to the LSA. From the right axillary access, a steerable sheath was advanced over an Amplatz wire to the origin of the ARSA. The fenestration was performed retrogradely using a 0.9 mm Turbo Elite laser catheter (Spectranetics). A 0.014 wire was inserted inside the fenestration and snared from the right CFA. Progressive dilatation of the fenestration was performed using a 2-mm cutting balloon followed by a 4-mm monorail balloon. The wire was exchanged for a 0.035 wire to advance an 8 L × 59 mm VBX stent, and deployed with 4 mm of the stent protruding into the aortic endograft. Postdilation of the stent was performed with a 12-mm balloon. After a failed attempt of closing the right axillary access with an Angio-Seal device (Terumo), a BeGraft peripheral stent was inserted from the femoral access passing inside the ARSA and deployed precisely at the axillary puncture site. Femoral Abbott Prostyles were closed successfully.

**Figure 2:**
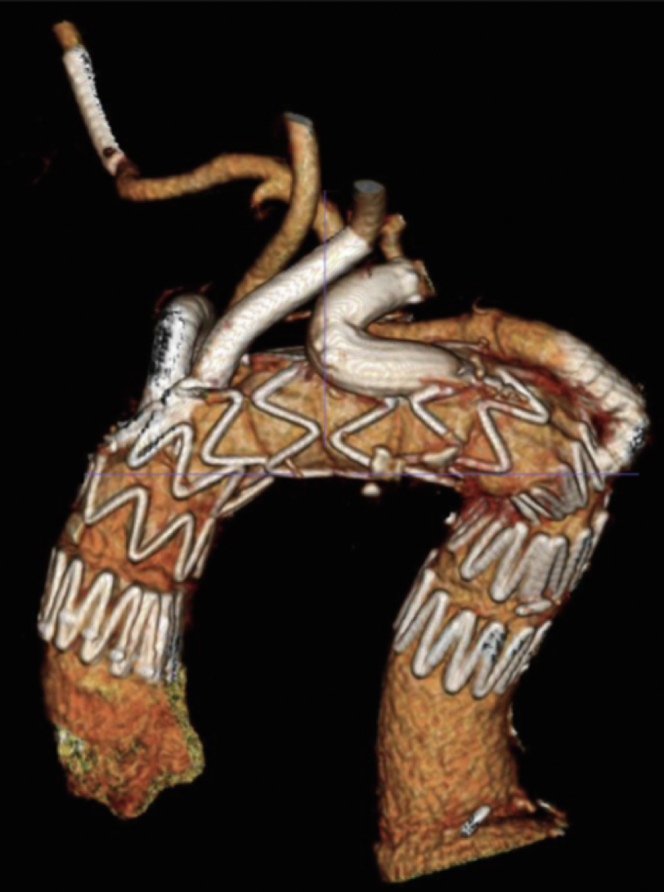
Postoperative 3-dimensional computed tomography angiography reconstruction of the 4-vessel aortic arch repair.

Total fluoroscopy time was 185 minutes with a total of 165 mL contrast used. The postoperative computed tomography angiography demonstrated no endoleaks or access-related complications (Figure 2). The postoperative course was unremarkable, and the patient was discharged home on postoperative day 5. At 11 weeks’ follow-up, the patient had no access-related or health-related complications.

## Discussion

This article described a fully percutaneous 4 vessels aortic arch repair using a 3 inner branch custom-made device complemented by 1 laser fenestration. Typically, surgical exposure of the carotid arteries is needed for retrograde catheterization of branches leading to the supra-aortic trunks. Totally percutaneous techniques have been developed to minimize access complications, especially in obese patients.^2^^,^^3^

Currently, companies do not produce custom-made devices for four supra-aortic vessels. In this case, deploying an additional distal fenestrated module for the ARSA was technically unfeasible due to the minimal distance between the LSA and ARSA, which would have resulted in an insufficient overlap and a high risk of a type III endoleak. We believed that in situ fenestration was the most reliable option to revascularize the ARSA.^4^

A total femoral approach for the 3-inner branches catheterization and bridging stents deployment was the ideal solution for this patient because cannulating the first branch via the right axillary was not an option. The absence of an innominate trunk allowed for a smaller diameter bridging stent for the first branch, enabling easier tracking from a femoral approach. BeGraft+ did have a good flexibility to reach the branch with a considerable radial force. Also, a retrograde orientation for the second branch would have facilitated easier catheterization and stent-tracking in the LCCA, although its long-term permeability could have been compromised, considering its acutely angled takeoff from the arch.

Mougin and colleagues^2^ were the first to perform a total percutaneous endovascular arch repair using the 3-vessel inner-branch device, followed by Tenorio and colleagues^5^ who report a total percutaneous transfemoral approach. To our knowledge, there are no published cases involving a totally percutaneous 4-vessel endovascular aortic arch repairs using a branched endograft.

## Conclusions

Four-vessel endovascular arch repair using a triple-inner branch stent-graft and 1 in situ laser fenestration can be safely performed using a complete percutaneous approach.

## Conflict of Interest Statement

Dr Haulon is a consultant for Bentley and Cook. Dr Charbonneau is a consultant for Cook. All other authors reported no conflicts of interest.

The *Journal* policy requires editors and reviewers to disclose conflicts of interest and to decline handling or reviewing manuscripts for which they may have a conflict of interest. The editors and reviewers of this article have no conflicts of interest.

## Supplementary Data

Video 1 Main stages of the procedure. Video available at: https://www.jtcvs.org/article/S2666-2507(24)00429-2/fulltext.
