# Supplementary figures and images for: Total percutaneous 4-vessel endovascular aortic arch repair with a triple inner-branch device and a fenestration
Source: JTCVS Tech. 2024 Oct 2;28:18–21. doi: 10.1016/j.xjtc.2024.09.020 (PMC11632385; doi:10.1016/j.xjtc.2024.09.020)

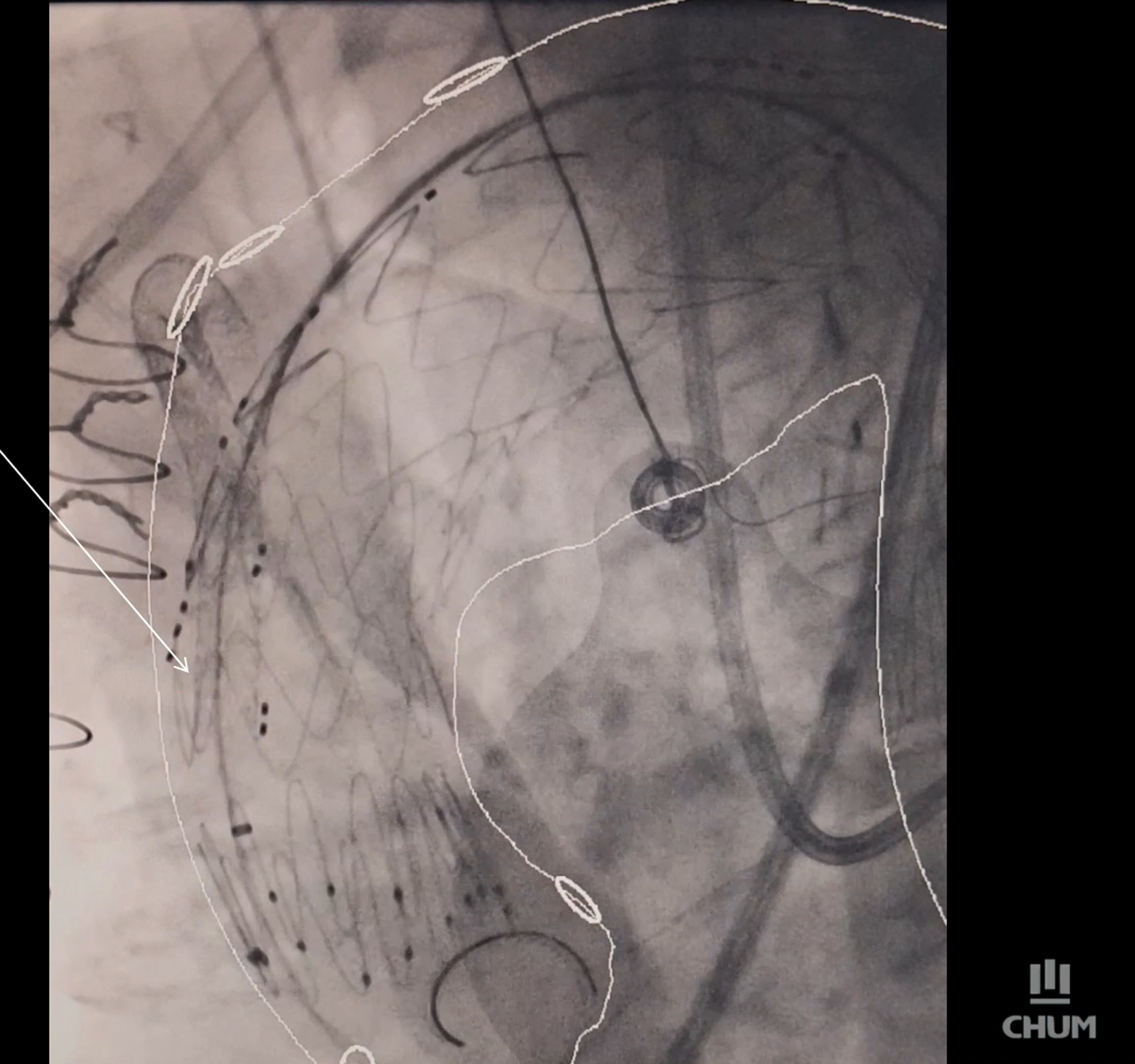

Supplement: Video 1 — Main stages of the procedure. Video available at: https://www.jtcvs.org/article/S2666-2507(24)00429-2/fulltext. [file fx2.jpg]
